# Conservation insights from wild bee genetic studies: Geographic differences, susceptibility to inbreeding, and signs of local adaptation

**DOI:** 10.1111/eva.13221

**Published:** 2021-03-25

**Authors:** Evan P. Kelemen, Sandra M. Rehan

**Affiliations:** ^1^ Department of Biology York University Toronto ON Canada

**Keywords:** climate change, commentary sex determination, conservation biology, habitat fragmentation, landscape genetics, wild bee

## Abstract

Conserving bees are critical both ecologically and economically. Genetic tools are valuable for monitoring these vital pollinators since tracking these small, fast‐flying insects by traditional means is difficult. By surveying the current state of the literature, this review discusses how recent advances in landscape genetic and genomic research are elucidating how wild bees respond to anthropogenic threats. Current literature suggests that there may be geographic differences in the vulnerability of bee species to landscape changes. Populations of temperate bee species are becoming more isolated and more genetically depauperate as their landscape becomes more fragmented, but tropical bee species appear unaffected. These differences may be an artifact of historical differences in land‐use, or it suggests that different management plans are needed for temperate and tropical bee species. Encouragingly, genetic studies on invasive bee species indicate that low levels of genetic diversity may not lead to rapid extinction in bees as once predicted. Additionally, next‐generation sequencing has given researchers the power to identify potential genes under selection, which are likely critical to species’ survival in their rapidly changing environment. While genetic studies provide insights into wild bee biology, more studies focusing on a greater phylogenetic and life‐history breadth of species are needed. Therefore, caution should be taken when making broad conservation decisions based on the currently few species examined.

## 1 INTRODUCTION

Bees are vital pollinators in ecosystems (Ollerton, 2017; Winfree et al., 2018) and have an estimated economic value of $235–577 billion annually (FAO, 2016). Therefore, worldwide declines in bee abundances pose a substantial conservation concern (Allen‐Wardell et al., 1998; Bartomeus et al., 2013; Freitas et al., 2009; Mathiasson & Rehan, 2020; Williams, 1982). The reasons for the declines are multifaceted, and researchers are just starting to uncover how anthropogenic threats, such as habitat degradation, fragmentation, and climate change, are affecting bee populations (Centrella et al., 2020; Durant & Otto, 2019; Glaum et al., 2017; Goulson et al., 2015). New molecular tools and analyses are making it possible to answer previously intractable conservation questions, providing insights into population connectivity, inbreeding depression, and local adaptation (Allendorf et al., 2010).

Genetic analyses are a valuable tool for bee conservation because bees are typically small and fast‐flying, making tracking individuals directly challenging. Genetic tools are important in conservation biology as they allow researchers to calculate many key parameters, like population structure, genetic diversity, and connectivity (Allendorf et al., 2007). These parameters can identify specific management units, distinct populations that should be monitored separately (Palsbøll et al., 2007; Waples & Gaggiotti, 2006). Isolated populations with low genetic diversity are of conservation concern as these populations often have lower mean fitness (Cameron et al., 2011; Frankham, 2015; Whitehorn et al., 2011, 2014).

New genetic tools are allowing researchers to understand how populations are responding to changes in the landscape. Studies have historically analyzed population structure by testing for differentiation among populations or isolation‐by‐distance, but now are increasingly incorporating landscape‐level data to create more realistic genetic models (Danforth et al., 2003; Jaffé, Castilla, et al., 2016; Lozier et al., 2013; Zayed et al., 2005). Landscape genetics identifies landscape features that structure a species’ genetic variation at the individual and population levels (Manel et al., 2003). The recent availability of spatially and temporally fine‐scale environmental data has given scientists the capabilities to identify the environmental factors that influence historical and contemporary population structures (Dellicour et al., 2017; Jaffé, Castilla, et al., 2016; López‐Uribe et al., 2015; Lozier et al., 2013). These techniques have even been combined with geographic distribution models based on climate projections to predict how species will respond in the future (Françoso et al., 2019). Understanding the spatial patterns of genetic diversity and the factors that impede gene flow in species, especially for rare species or those with isolated populations, is vital for their management and, ultimately, their long‐term survival (Hoffmann et al., 2015).

Many of the obstacles that species of conservation concern face are overcome by invasive species. Invasive species are exhibit isolated populations with low genetic diversity (Dlugosch & Parker, 2008). Genetic studies on these invaders can inform researchers about the genetic diversity necessary for populations to persist (Schmid‐Hempel et al., 2007; Zayed et al., 2007) and the effects of human‐mediated movement on population structure (Strange et al., 2017). Scientists could leverage this information when making management decisions.

As next‐generation sequencing costs have decreased, some studies have shifted from using tens of loci to thousands, while others have even implemented full genome resequencing. This shift has given analyses more power to detect outlier loci and perform genotype by environment analyses (Jackson et al., 2020; Jaffé et al., 2019; Theodorou et al., 2018). Landscape genomics studies focus on identifying genes under selection (Storfer et al., 2018). By leveraging thousands of genetic markers across genomes (Baird et al., 2008), these large datasets can identify neutral loci, which can provide a more precise understanding of gene flow, and candidate loci that indicate possible local adaptations (Jackson et al., 2020; Jaffé et al., 2019; Theodorou et al., 2018). These techniques are important for bee conservation as they identify how species are responding to environmental stressors and potential adaptative alleles within populations and are already assisting forestry restoration initiatives (Carvalho et al., 2019; Jaramillo‐Correa et al., 2015).

**FIGURE 1:**
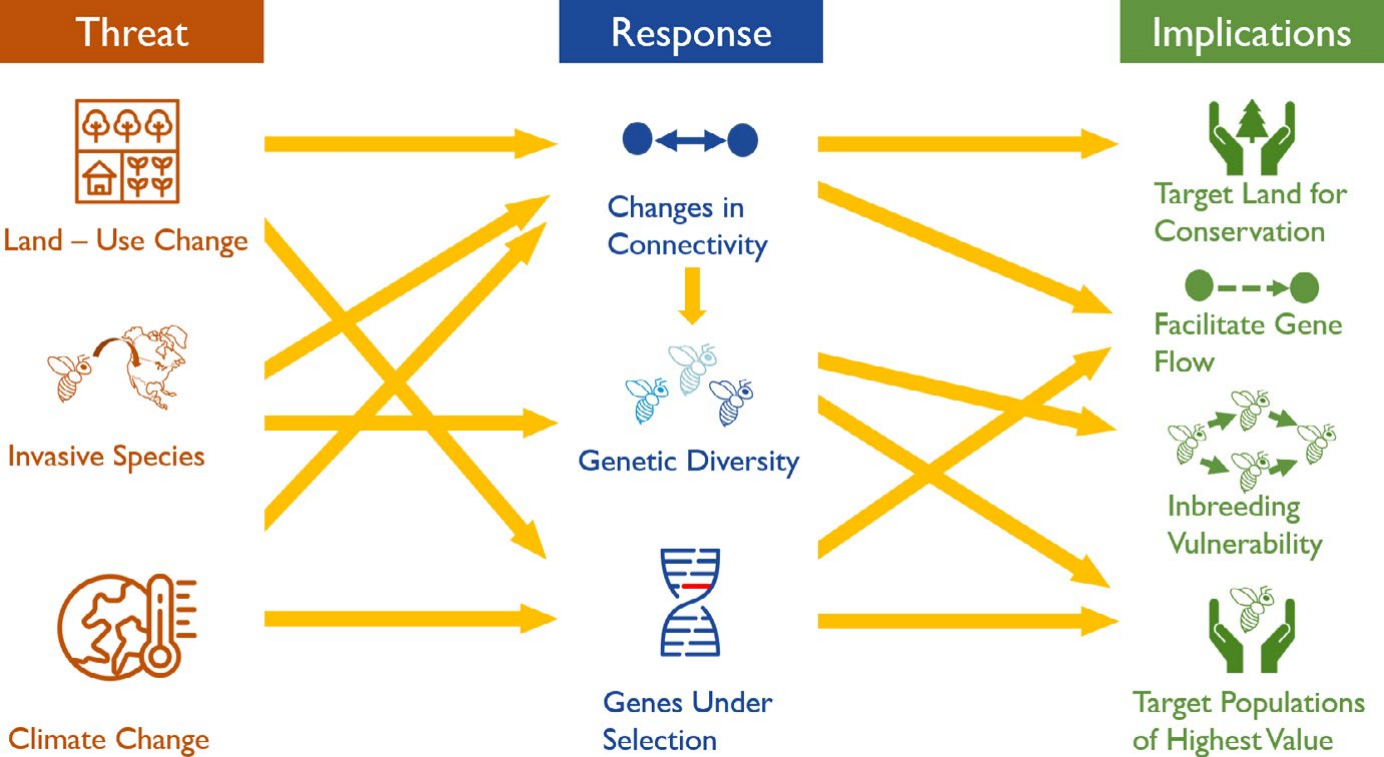
Recent genetic tools provide insights into the response of wild bees to anthropogenic threats and their implications for conservation efforts. This review synthesizes interconnected topics (indicated by the arrows), focusing on potential geographic changes in connectivity, insights from invasive species, and advances gained from exploring genes under selection

This review aims to highlight how recent genetic tools have advanced our understanding of wild bees and the potential implications for their conservation (Figure 1). Landscape genetic and genomic research, along with traditional population genetic studies, are important to understand how wild bees respond to anthropogenic threats. This review emphasizes recent findings, including (i) the potential geographic differences in the genetic response of bees to land‐use change, (ii) insights from documented invasion biology, (iii) signs of selection and local adaptation, and (iv) recent advances and future challenges.

## 2 POTENTIAL GEOGRAPHIC DIFFERENCES IN GENETIC RESPONSE

A working hypothesis is that temperate species are more sensitive than tropical species to natural and human changes in land‐use threats (Landaverde‐González et al., 2017). This potential difference in sensitivity could be due to life‐history traits associated with temperate and tropical species such as flight season, dispersal ability, or population size (De Palma et al., 2015; Michener & Amir, 1977). Additionally, it could be due to taxonomic differences in the bees often studied in these regions. Studies from both regions have focused mainly on corbiculate bees, but temperate studies have primarily emphasized bumble bees (Goulson et al., 2011; Jha, 2015; Lozier, 2014), while tropical studies have predominantly investigated orchid and stingless bees (Jaffé, Pope, et al., 2016; Zimmermann et al., 2011). Also, many of the earlier studies used microsatellites, which may not provide the resolution needed to detect more subtle populations structure that higher density markers like RAD sequencing can detect (Jaffé, Pope, et al., 2016; Jaffé et al., 2019). It is important to understand if tropical species, but not temperate species, are able to maintain population connectivity in the face of landscape‐level changes. This potential difference between temperate and tropical species would suggest the need for geographically specific conservation actions.

Temperate species appear sensitive to landscape changes (Goulson et al., 2011; Jha & Kremen, 2013b; López‐Uribe et al., 2015; Lozier et al., 2013). Across the human‐altered landscape, temperate bee species show reduced nesting density, limited gene flow, and marked population differentiation (Darvill et al., 2010; Ellis et al., 2006; Jha & Kremen, 2013b). While genetic structuring may be weak or absent at the continental level (Lozier et al., 2011; Maebe et al., 2019), genetic structuring occurs at small spatial scales at distances as little as a kilometer (Davis et al., 2010; Goulson et al., 2011; Jha & Kremen, 2013b). For instance, the North American yellow‐faced bumble bee, *Bombus vosnesenskii*, shows near panmixia across its current range at large spatial scales but exhibits regional structuring from urbanization limiting gene flow (Jha & Kremen, 2013b). It is unclear if such local structure persists or is especially relevant for the overall species’ genetic diversity in the long run. However, this genetic differentiation can occur over relatively short periods, in as little as a few months to a year (Jha, 2015). Urbanization also increases isolation in a solitary ground‐nesting bee, *Colletes floralis* (Davis et al., 2010), and increases inbreeding in the congener *C. inaequalis* (López‐Uribe et al., 2015). Ground nesters are particularly vulnerable to land‐use change as any changes that compact or till the soil limit available nesting sites (Jha & Kremen, 2013a). Gene flow may be maintained with the help of available floral resources. In the ruderal bumble bee, *B. ruderatus*, in New Zealand, areas separated by poor forage are significantly differentiated (Bartlett et al., 2016). Restoring habitat by sowing flower patches within an intensive agricultural landscape appears to be enough to maintain gene flow in bumble bees (Dreier et al., 2014). Land‐use changes are not detrimental to all bee species. The eastern carpenter bee, *Xylocopa virginica*, shows increased gene flow across human‐altered environments compared to semi‐natural areas, likely due to this species nesting in residential hardwood structures (Ballare & Jha, 2020).

In contrast to temperate species, tropical bee species appear more robust to landscape changes. Across nine species of orchid bees, *Euglossa spp*., there was no effect of recent deforestation on genetic differentiation (Cerântola et al., 2011; Soro et al., 2017; Suni, 2017; Zimmermann et al., 2011). Similarly, land‐use did not affect gene flow in seventeen stingless bee species (Jaffé, Pope, et al., 2016; Landaverde‐González et al., 2017; however, see Jaffé et al., 2019). In fact, despite recent habitat fragmentation, it appears that one species of stingless bee, *Trigona spinipes*, has gone through a recent population expansion. A population expansion may have occurred because this species travels well across the mosaic landscape of forest fragments and coffee plantations as inferred from the little genetic differentiation at a scale of 200 km (Jaffé, Castilla, et al., 2016). Repeatedly, genetic studies have suggested tropical species are remarkable dispersers (Jaffé, Castilla, et al., 2016; Landaverde‐González et al., 2017; Soro et al., 2017), but actual dispersal observations have failed to show that tropical species travel further than temperate species (Kraus et al., 2009; Roubik & Aluja, 1983; Wikelski et al., 2010). Therefore, it is unclear why tropical species appear to have more panmictic populations. Regardless of the reason, current changes in land‐use do not appear to impede gene flow or cause any genetic differentiation in tropical bee species (Jaffé, Pope, et al., 2016; Landaverde‐González et al., 2017; Soro et al., 2017; Suni, 2017; Zimmermann et al., 2011).

Geographic differences may reflect temporal differences in land‐use. The temperate areas have a long history of deforestation, with the majority of it occurring centuries ago, while deforestation in the tropics is relatively more recent occurring within the last century (Williams, 2003). The loss of genetic diversity can be a slow process (Jackson & Fahrig, 2014). Simulations suggest a time lag between the introduction or removal of a dispersal barrier and the ability to detect it using landscape genomics (Epps & Keyghobadi, 2015; Landguth et al., 2010). The length of time lag will depend on the nature of landscape change, a species’ dispersal ability, and its effective population size (Landguth et al., 2010; Latter, 1973; Nei, 1977). For instance, genetic diversity in alpine butterflies reflected spatial patterns of forest cover from 40 years in the past rather than contemporary forest cover (Keyghobadi et al., 2005). Therefore, it may not be that tropical species are more robust to landscape changes but that these changes are only just starting to affect these species.

There is evidence from tropical bumble bees suggesting that the genetic diversity of tropical species is changing. In South America, the genetic diversity of the bumble bee *B. pauloensis* has decreased since the 1950 s (Maebe et al., 2018). Also, while *B. ephippiatus* shows no genetic patterns of isolation‐by‐distance, some differentiation is starting to occur due to recent deforestation (Landaverde‐González et al., 2018). *B. huntii* shows genetic structuring in southern Mexico related to its distribution across different high‐elevation montane habitats, structure not observed at larger spatial scales in North America (Koch et al., 2018). Additionally, fine‐scale genetic structuring may be common in stingless bees as well, but the resolution provided by microsatellites allowed previous studies to detect only strong effects of landscape features. Jaffé et al., (2019) found weak but significant structuring in *Melipona subnitida* by employing thousands of genetic markers using RAD sequencing (Jaffé et al., 2019). These early signs of structuring support the idea that there may be a time lag between land‐use changes and detectable changes of genetic diversity.

The difference between temperate and tropical species could be an artifact of geographic differences in the landscape features examined. Most tropical species are examined for differentiation due to deforestation for agriculture (Soro et al., 2017; Suni, 2017; Zimmermann et al., 2011), whereas studies on temperate species have included urban landscapes (Davis et al., 2010; Jha & Kremen, 2013b; López‐Uribe et al., 2015). When examining just the effect of agriculture on gene flow in the temperate bumble bee species *B. pascuorum*, researchers found no effect of landscape on population structure (Herrmann et al., 2007). However, urban land‐use has been included in some analyses of tropical species and was found not to significantly structure the population (Landaverde‐González et al., 2017).

Genetic differences between temperate and tropical species also may be revealing additional historic environmental differences. For any given species, the locations of suitable habitats and barriers to dispersal have changed over periods of global warming and cooling. Differences in genetic diversity among European bumble bees existed before notable decreases in populations (Maebe et al., 2016). Instead, differences in genetic diversity were likely due to bottlenecks from glaciation (Wallberg et al., 2014). RAD sequencing identified the Iberian Peninsula as a potential glacial refugium for *B. terrestris* (Silva et al., 2020). This important pool of genetic diversity reflects the importance of long‐term influences that may mask more recent effects from anthropogenic factors. Coalescent simulations suggest that *B. hortorum* and *B. pascuorum* range shrank along with *B. terrestris* during periods of cooling, and populations became isolated due to fragmentation of suitable habitat (Dellicour et al., 2017). Therefore, present‐day structuring among populations may be explained by past barriers to dispersal (Miranda et al., 2017). Conversely, the absence of current barriers to dispersal may explain population structuring. For instance, the bumble bee species, *B. hortorum*, likely traveled between shallow sea islands in Scotland during the last ice age when sea levels were lower (Goulson et al., 2011). Genetic diversity is also linked not only to the presence of suitable habitat but the stability of the habitat through time. In North America, the genetic diversity of *B. huntii* is related to environmental niche stability (how much a location was predicted to have changed through time). Since the last glacial maximum, the more unstable the niche is at a location, the more genetically diverse the population (Koch et al., 2018). Therefore, the survivability of a species is influenced by past environments as well as its current environment.

Taken together, understanding the historical and contemporary patterns driving species‐level differences in diversity will help researchers identify which species are of most concern. If tropical bee species are more robust to environmental disruption, then conservation efforts should prioritize temperate species. Researchers should try to understand what makes temperate species more vulnerable to landscape changes. However, tropical species may not be more robust. The lack of population structuring may be due to historical differences in land‐use or the species examined. Therefore, it is essential to monitor more diverse species with high‐density markers to identify even subtle population structures. This monitoring will also help researchers understand how quickly genetic changes reflect landscape changes if there is a delay.

## 3 INSIGHTS FROM DOCUMENTED INVASION BIOLOGY

Invasive species can provide useful insights into how species respond when they encounter novel climatic and biotic selective pressures (Moran & Alexander, 2014). Invasive species also face many of the demographic perturbations faced by native species in fragmented landscapes of population bottlenecks or range expansions following land‐use change (Dlugosch & Parker, 2008; Moran & Alexander, 2014; Zayed et al., 2007). Therefore, scientists can gain valuable insights from invasive bee species about the genetic variation and gene flow needed to sustain native bee populations and the effects of human‐mediated translocations. Invasive bees are also detrimental to local pollinators (Graham et al., 2019; LeCroy et al., 2020; Morales et al., 2013) and can disrupt pollination services provided by local bees (Morales et al., 2017). Molecular techniques can also help identify how these invaders may be impacting native species. All these studies together can help inform management plans.

It is posited that bees should be sensitive to low population sizes (Zayed, 2009), but evidence from empirical studies from invasive species suggests that this may not be the case (Schmid‐Hempel et al., 2007; Zayed et al., 2007). As haplodiploid organisms, bees can purge deleterious alleles through haploid males, making them relatively immune to inbreeding compared to diploid organisms (Hedrick & Parker, 1997; Luna & Hawkins, 2004). However, their assumed sex‐determination system, a single‐locus complementary sex determination (CSD), theoretically imposes substantial genetic load through homozygotes at the sex locus resulting in sterile diploid males (Beye et al., 2003; Cook & Crozier, 1995). Since Hymenoptera fertilize their eggs to produce females, the production of these diploid males effectively increases female mortality and reduces potential population growth (Stouthamer et al., 1992). Diploid male production theoretically initiates a rapid “extinction vortex” that elevates the extinction rate of haplodiploid organisms by an order of magnitude greater than diploid organisms (Zayed & Packer, 2005). However, invasive bee species have revealed that populations can persist with low genetic diversity. Accidental introductions of invasive bees are often started by a few individuals (Schmid‐Hempel et al., 2007; Zayed et al., 2007). *Lasioglossum leucozonium*, a solitary ground‐nesting bee, went through a severe bottleneck in its introduction to North America from Europe, possibly established by a single mated female (Zayed et al., 2007). This low genetic diversity has resulted in 30% of female destined eggs producing diploid males, a sign of inbreeding in Hymenoptera (Zayed et al., 2007). However, despite this severe genetic load, this bee is found in large numbers across its range (Atwood, 1933; Bushmann & Drummond, 2015; Mathiasson & Rehan, 2019; Moisan‐DeSerres et al., 2015). In Tasmania, the European bumble bee, *B. terrestris*, has been very successful despite its low genetic diversity (Schmid‐Hempel et al., 2007). These populations could have been founded by as many as two individuals from New Zealand back in 1991 (Schmid‐Hempel et al., 2007).

This low genetic diversity has not led to a drastic increase in diploid males or the predicted “extinction vortex” associated with bees’ single‐locus CSD system (Zayed & Packer, 2005). While the ancestral state of Hymenoptera is proposed to be single‐locus CSD (Asplen et al., 2009), the classification of individuals possessing such a system is often based on the presence of diploid males and biased sex ratios during inbreeding experiments (Asplen et al., 2009; Van Wilgenburg et al., 2006). It is possible that in bees, multiple mechanisms of sex determination have evolved. In hymenopterans, three mechanisms are known, the single‐locus CSD in honey bees *Apis mellifera* (Hasselmann et al., 2008), multi‐locus CSD in the parasitoid wasp *Lysiphlebus fabarum* (Matthey‐Doret et al., 2019), and parental genome imprinting in the jewel wasp *Nasonia vitripennis* (Zou et al., 2020). An alternative mechanism to single‐locus CSD may explain why diploid males are at lower levels than expected in some inbred wild bee populations (Boff et al., 2014).

Research on invasive bees also suggests that moving bees may not harm population‐wide genetic diversity. In North America, the alfalfa leafcutter bee, *Megachile rotundata*, is currently being propagated and managed commercially for its pollination services. It has genetic diversity similar to its native European populations but has little genetic structuring in its introduced range. The lack of structuring is likely due to the large portion of *M. rotundata* purchased from Canada and moved among farms in the United States. This movement has created a near panmictic *M. rotundata* population maintaining high genetic diversity (Strange et al., 2017). It is unclear if this human‐mediated admixture will prevent populations from adapting to local environmental variation or climate change, though this appears not to be the case in other animal systems (Fitzpatrick et al., 2020; Poirier et al., 2019; Rick et al., 2019). While studies of invasive species are many, data remain scarce for native bee ranges and restoration efforts. To date, there appear to be no reports of facilitated gene flow and only two attempts to reintroduce native bees through translocations. The movement *B. subterrananeus* from Sweden to England has been unsuccessful (Gammans, 2020), but the movement of *Hylaeus anthracinus* within Hawaii to restored sites has been successful (Magnacca, 2020). From a conservation standpoint, current studies suggest that reintroducing bees may be possible while maintaining genetic diversity; however, any translocations should weigh all potential costs and benefits.

The introduction of bees for pollination services can have devastating results. Over 15 years, the invasive mason bee species *Osmia taurus* has increased 800% across the mid‐Atlantic United States, while six native mason bee species have decreased by 76–91% (LeCroy et al., 2020). Honey bees with *A. mellifera scutellata* ancestry, also known as Africanized honey bees, are associated with local extinctions (Portman et al., 2017) and behavioral changes of native pollinators (Roubik & Villanueva‐Gutierrez, 2009). These honey bees came to dominate across most of the Americas after escaping in 1957 from managed colonies in Brazil (Winston, 1992). Genomic analysis indicates no substantial reduction in genetic diversity associated with this scutellata ancestry despite its rapid expansion suggesting a competitive fitness advantage at lower latitudes than honeybees of European ancestry (Calfee et al., 2020). However, there is a wide hybrid zone between these two ancestries, suggesting that honey bee ancestry tracks environmental variables. Even when kept within a greenhouse, non‐native pollinators can escape (Morandin et al., 2001). Escaped *B. terrestris* are hybridizing with native populations, as indicated by the introgression of alleles from commercial subspecies into the local subspecies on the Iberian Peninsula (Seabra et al., 2019). The introduction of these pollinators also harms native species due to their associated pathogens. Pathogen spillover from commercial honey bee and bumble bee colonies may have caused declines in *B. terricola*. Population genomic work indicated recent declines in this species’ effective population size and positive selection on several immune genes, suggesting it may be experiencing pressure from a novel pathogen (Kent et al., 2018). The spread of *B. terrestris* and its associated parasite *Crithidia bombi* across South America is linked to the disappearance of *B. dahlbomii* (Schmid‐Hempel et al., 2014). Declines in the blue orchard bee, *O. lignaria*, may also be due to trans‐continental movement of pathogens associated with the closely related and invasive *O. cornifrons* (Bartomeus et al., 2013; Hedtke et al., 2015). The spread of parasites is a conservation concern because bee species and populations with lower genetic diversity are disproportionately affected (Lattorff et al., 2016; Parsche & Lattorff, 2018; Whitehorn et al., 2014). Populations with lower genetic diversity are known to have higher parasite prevalence (Parsche & Lattorff, 2018; Whitehorn et al., 2011, 2014). This relationship suggests that already vulnerable bee populations are even more susceptible to invasive species and their associated parasites.

## 4 SIGNATURES OF SELECTION AND LOCAL ADAPTATION

Genetic tools can identify associations between population genetic and spatial patterns that may indicate selection. Understanding how populations respond to their local climate is critically important for conservation (Franks & Hoffmann, 2012). As anthropogenic threats alter local environments, it is vital to maintain a species’ evolutionary potential by preserving as much genetic variation as possible (Hoffmann & Sgro, 2011; Sgrò et al., 2011). Studies have traditionally used population structuring based on allele frequencies, measured as F_st_, to detect potential signatures of selection from the local environment (Guo et al., 2016; Pujolar et al., 2014). With the advent of next‐generation sequencing, studies have the power for more advanced environmental association analyses (Jackson et al., 2020; Jaffé et al., 2019; Theodorou et al., 2018). Environmental association analyses are often used in concert with the more traditional analyses, and these two approaches are complementary. Landscape genomics enhances our ability to identify potential genes under selection. There are several types of environmental association analyses, and each control for demographic structuring within species in different ways (Rellstab et al., 2015). Therefore, when testing for signatures of selection, it is beneficial to combine multiple methods to identify top candidate outliers associated with environmental conditions.

Within bees, few studies have investigated patterns of genetic differentiation according to their local environment. The studies that have performed such fine‐scale analyses have only investigated these patterns in social species. In *B. vosnesenskii* and *B. vancouverensis*, there is an association between temperature and genes related to neural and neuromuscular function and ion transport (Jackson et al., 2020). These loci may be under selection to maintain neural and muscle tissue under extreme temperatures. This study also found an association between precipitation and genes related to cuticle formation, homeostasis, and tracheal and respiratory system development. These loci may be under selection to prevent desiccation in drier habitats (Jackson et al., 2020). There is also putatively adaptive genetic variation associated with latitude in the stingless bee *Melipona subnitida* (Jaffé et al., 2019) and the honey bee *A. melliferia* (Hadley & Betts, 2012; Henriques et al., 2018). Iberian Peninsula populations of *A. melliferia* show latitudinal gradients associated with clock genes, suggesting that the circadian rhythm is involved in local adaptation (Henriques et al., 2018). These species also show distinct adaptive genetic variation along elevational gradients (Jaffé et al., 2019; Wallberg et al., 2017). East Africa populations of *A. melliferia* exhibited panmixia except for two loci that exhibited near fixation for a highland and lowland haplotype (Wallberg et al., 2017). These loci occurred in octopamine receptor genes, which have a role in foraging and learning. Knowing what environmental factors cause populations to differ can inform conservation efforts. As individuals move with climatic conditions, alleles previously beneficial in a population may be lost due to an influx of migrants (Slatkin, 1987). For instance, in *M. subnittida*, as temperature increases due to climate change, lowland populations may move to higher elevations. However, this poses a problem for highland populations (Jackson et al., 2018; Rubidge et al., 2012). Recurrently, as bee populations find refuge in higher elevations (Marshall et al., 2020; Nooten & Rehan, 2020; Tucker & Rehan, 2017), these populations are becoming isolated, potentially further contributing to their declines (Cameron et al., 2011). Therefore, to retain the evolutionary potential of high‐elevation species, conservation efforts should focus on these most vulnerable populations. It may be important to facilitate gene flow among mountain top refugia or possibly translocate populations into habitats where favorable alleles are better suited for local environmental conditions (Aitken & Whitlock, 2013).

Signatures of selection have also been found associated with urban environments (Theodorou et al., 2018). Overall there is low differentiation between urban and rural sites in the red‐tailed bumble bee, *B. lapidaries* (Theodorou et al., 2018). This low differentiation may have to do with the time scale under which selection has had to act. Most other studies focus on long‐term adaptations (e.g., climate variables), but the response to urbanization is a more recent and open question. However, it appears that there are some signs of selection in *B. lapidaries* (Theodorou et al., 2018). There seem to be divergent allele frequencies between urban and rural sites in genes associated with molecular binding and metabolic processes (Theodorou et al., 2018). These genes are related to responses to environmental stress, such as heat‐stress and oxidative‐stress, which are likely adaptations to the stress of the urban environment (urban warming, pollution, parasites, and costly foraging) (Isaksson, 2015). While urbanization may exert similar selective pressures on other bee species, more studies are needed to confirm these findings generalizability. The reducing costs of next‐generation sequencing are opening up the possibilities to identify specific loci under selection across populations and species. Few studies currently exist exploring signatures of selection, and these have largely focused on bumble bees; however, there are many open questions across the broad diversity of wild bees, including the vast majority of understudied genera.

## 5 RECENT ADVANCES AND FUTURE CHALLENGES

Advances in genetic methods, including reduced representation genomics tools, provide cost‐effective ways to increase statistical power in recent studies. While genome‐scale genetic studies have only been accessible to well‐funded model systems, reduced representation genomic techniques such as RAD sequencing can provide thousands of single nucleotide polymorphisms (SNPs) (Lecocq et al., 2013; Lozier, 2014). This abundance of SNPs gives researchers the power to revisit study systems to determine if the absence of population structuring observed is an artifact of the limited number of previously available markers (Lozier, 2014). Along with population structuring, candidate loci under selection can be determined from outlier analyses (Jackson et al., 2020; Theodorou et al., 2018). These analyses identify loci that exhibit significantly higher or lower among‐population genetic differentiation than expected under neutrality. However, the outliers determined by outlier analyses may result from selection on nearby parts of the genome rather than the gene or region associated with any given locus. Without a reference genome, as is the case with many bee species, genome‐wide patterns and selective sweeps cannot be detected, which raises the rate of false negatives (Hoban et al., 2016). Additionally, reduced representation techniques that sequence parts of the genome, like RAD sequencing, may incompletely sample the genome. The sparseness of markers may fail to detect adaptive loci when linkage disequilibrium is short (Lowry et al., 2017). It is also important to consider that regions within the genome experience different recombination rates with lower rates leading to above‐average genetic differentiation, such as in centromeric regions (Cruickshank & Hahn, 2014). This difference in recombination rates can potentially lead to bias in detecting SNPs based on the gene's location. Reasonable estimates of linkage disequilibrium and chromosome size are missing for the vast majority of species; therefore, researchers should maximize the number of polymorphic markers to alleviate these concerns. While every set of molecular markers has its potential biases, RAD sequencing is still a powerful method when whole‐genome sequencing is unavailable since the loci are thought to be randomly distributed throughout the genome (Cariou et al., 2016).

More whole genomes are becoming available to leverage additional information from genetic markers to address the aforementioned issues. Currently, there are 53 published bee genomes (Table S1). Most of these are restricted to the family Apidae (45), but genomes from Megachilinae (3), Halictidae (4), and Colletidae (1) have been published. Also, ongoing efforts by international organizations such i5 k (http://i5k.github.io/) and the Tree of Life Program (https://www.darwintreeoflife.org/) are sure to contribute more genomes.

Other high throughput omics approaches (transcriptomics, proteomics, metabolomics, metagenomics, phenomics, etc.) can increase scientists’ abilities to identify the molecular mechanisms underpinning a species’ responses to their environment (Carducci et al., 2020; Vieira et al., 2021; Voelckel et al., 2017). Scientists can identify putative local adaptations by identifying variation in the expression of biomolecules associated with environmental factors (Voelckel et al., 2017). In *B. vosnesenskii*, transcriptomes approaches have identified co‐expressed gene sets that correlate with intrapopulation differences in cold tolerance (Pimsler et al., 2020). The population‐specific phenotype and genotype are important to consider for species management (Lozier et al., 2015). Therefore, when integrated with landscape data, these omics methods create a more holistic understanding of species, which can be translated into management plans and policies (Connon et al., 2018). Comparing findings between species is an ongoing challenge. Mutation rates between species may differ (however, see Liu et al., 2017), and species are known to have different genome sizes (Kapheim et al., 2015), gene family expansions (Simola et al., 2013), and recombination rates (Jones et al., 2019). Furthermore, the phylogenetic coverage of species is expanding but remains focused in a few genera (*Bombus* (Jha, 2015; Lozier, 2014; Maebe et al., 2016), *Euglossa* (Soro et al., 2017; Suni, 2017; Zimmermann et al., 2011)). There are efforts to study nonmodel bee species, including using museum species to develop genotype and genomic data for bees (Vaudo et al., 2018). Many bee genera may respond differently, and there is a large gap in knowledge and need to study noncorbiculate bees (Apidae: Apinae). Additionally, researchers need to sample bees with more diverse lifestyles, including the vast majority of solitary species. Most of the bee species included in genetic studies are social (Jaffé, Castilla, et al., 2016; Maebe et al., 2018). However, sociality provides unique complexities and responses to anthropogenic change. Social species are known to have smaller effective population sizes (Chapman & Bourke, 2001), longer active seasons (Ogilvie & Forrest, 2017), and unique selective pressures (Field & Toyoizumi, 2020). While many social bees are important commercial pollinators, of the >20,400 bee species, only 9.4% are social (Danforth et al., 2019; Michener, 2007). As studies begin to examine solitary species, researchers will be better equipped to support their conservation. Additional functional traits may make species more vulnerable to anthropogenic threats. For instance, oligolectic or specialist bees are thought to have lower genetic diversity (Packer et al., 2005; Zayed et al., 2005). Therefore, by better understanding how the local habitat and dietary breadth of native bees affect their genetic diversity, researchers can inform better management plans to support their biodiversity.

As researchers learn more about how landscape shapes population structure and connectivity, new questions have arisen. Tropical species currently appear more robust than temperate species to anthropogenic changes (Jaffé, Pope, et al., 2016; Jha, 2015; López‐Uribe et al., 2015; Zimmermann et al., 2011). However, this may be due to differences in the species examined or temporal differences in land‐use (Goulson et al., 2011; Jaffé, Pope, et al., 2016; Jha, 2015; Lozier, 2014; Zimmermann et al., 2011). As deforestation in the tropics increases, will there be a subsequent decrease in bee genetic diversity? If genetic diversity and connectivity between populations remain, it raises the question of how do native bees maintain connectivity in fragmented habitats, and will this connectivity translate to demographic stability? Invasive bee species suggest that populations can persist with low genetic diversity and that commercialization and artificially moving bees may not necessarily reduce standing genetic diversity. The movement of individuals, potentially with beneficial genotypes, may be one method for conservation. Landscape genomic techniques are just starting to be adopted in bee conservation genetics and will provide insights into the genes under selection. Once candidate genes are identified, functional analyses are necessary next steps to confirm fitness effects. While reduced representation genomic sequencing tools are an important start to answering these questions, whole‐genome sequencing is increasingly affordable and will provide greater resolution in future studies. Lastly, to fully understand how anthropogenic changes impact bees more broadly and to fully inform the best course of conservation action, researchers need to study a wider evolutionary and ecological variety of bee species.

## CONFLICT OF INTEREST

None declared.

## Supporting information

Table S1

## Data Availability

Data sharing is not applicable to this article as no new data were created or analyzed in this study.
