# Supplementary material for: Conservation insights from wild bee genetic studies: Geographic differences, susceptibility to inbreeding, and signs of local adaptation
Source: Evol Appl. 2021 Mar 25;14(6):1485–96. doi: 10.1111/eva.13221 (PMC8210791; doi:10.1111/eva.13221)
Supplement: Supplementary file 1 — Table S1 [file EVA-14-1485-s001.docx]

Table S1: List of published and publicly available bee genomes

| **Family** | **Genus** | **Species** | **NCBI BioProject** | **Citation** |
| --- | --- | --- | --- | --- |
| Apidae | *Habropoda* | *laboriosa* | [PRJNA339959](https://www.ncbi.nlm.nih.gov/bioproject/PRJNA339959) | Kapheim et al., 2015 |
|  | *Apis* | *mellifera* | PRJNA471592 | Wallberg et al., 2019 |
|  | *Apis* | *cerana* | [PRJNA324433](https://www.ncbi.nlm.nih.gov/bioproject/PRJNA50293) | Park et al., 2015 |
|  | *Apis* | *laboriosa* | [PRJNA647849](https://www.ncbi.nlm.nih.gov/bioproject/PRJNA647849) | Lin et al., 2020 |
|  | *Apis* | *dorsata* | [PRJNA232132](https://www.ncbi.nlm.nih.gov/bioproject/PRJNA232132) | Oppenheim et al., 2020 |
|  | *Apis* | *florea* | PRJNA45871 | Qu et al., 2019 |
|  | *Bombus* | *terrestris* | [PRJNA68545](https://www.ncbi.nlm.nih.gov/bioproject/PRJNA68545) | Sadd et al., 2015 |
|  | *Bombus* | *vancouverensis* | [PRJNA623917](https://www.ncbi.nlm.nih.gov/bioproject/PRJNA623917) | Heraghty et al., 2020 |
|  | *Bombus* | *haemorrhoidalis* | PRJNA659133 | Sun et al., 2021 |
|  | *Bombus* | *pyrosoma* | PRJNA659133 | Sun et al., 2021 |
|  | *Bombus* | *turneri* | PRJNA659133 | Sun et al., 2021 |
|  | *Bombus* | *cullumanus* | PRJNA659133 | Sun et al., 2021 |
|  | *Bombus* | *sibiricus* | PRJNA659133 | Sun et al., 2021 |
|  | *Bombus* | *picipes* | PRJNA659133 | Sun et al., 2021 |
|  | *Bombus* | *consobrinus* | PRJNA659133 | Sun et al., 2021 |
|  | *Bombus* | *soroeensis* | PRJNA659133 | Sun et al., 2021 |
|  | *Bombus* | *polaris* | PRJNA659133 | Sun et al., 2021 |
|  | *Bombus* | *difficillimus* | PRJNA659133 | Sun et al., 2021 |
|  | *Bombus* | *confusus* | PRJNA659133 | Sun et al., 2021 |
|  | *Bombus* | *opulentus* | PRJNA659133 | Sun et al., 2021 |
|  | *Bombus* | *skorikovi* | PRJNA659133 | Sun et al., 2021 |
|  | *Bombus* | *superbus* | PRJNA659133 | Sun et al., 2021 |
|  | *Bombus* | *vosnesenskii* | PRJNA611634 | Heraghty et al., 2020 |
|  | *Bombus* | *bifarius* | [PRJNA623924](https://www.ncbi.nlm.nih.gov/bioproject/PRJNA623924) | Heraghty et al., 2020 |
|  | *Bombus* | *waltoni* | PRJNA659133 | Sun et al., 2021 |
|  | *Bombus* | *ignitus* | PRJNA659133 | Sun et al., 2021 |
|  | *Bombus* | *impatiens* | PRJNA61101 | Sadd et al., 2015 |
|  | *Bombus* | *breviceps* | PRJNA659133 | Sun et al., 2021 |
|  | *Ceratina* | *australensis* | [PRJNA302037](https://www.ncbi.nlm.nih.gov/bioproject/PRJNA302037) | Rehan et al., 2018 |
|  | *Ceratina* | *calcarata* | PRJNA340002 | Rehan et al., 2016 |
|  | *Ceratina* | *japonica* | PRJNA413373 | Shell et al., 2021 |
|  | *Ctenoplectra* | *terminalis* | PRJNA526224 | Shell et al., 2021 |
|  | *Eufriesea* | *mexicana* | [PRJNA339561](https://www.ncbi.nlm.nih.gov/bioproject/PRJNA339561) | Kapheim et al., 2015 |
|  | *Euglossa* | *dilemma* | [PRJNA388474](https://www.ncbi.nlm.nih.gov/bioproject/PRJNA388474) | Brand et al., 2017 |
|  | *Exoneura* | *robusta* | PRJNA413974 | Shell et al., 2021 |
|  | *Exoneurella* | *tridentata* | PRJNA526241 | Shell et al., 2021 |
|  | *Frieseomelitta* | *varia* | PRJNA528016 | de Paula Freitas et al., 2020 |
|  | *Heterotrigona* | *itama* | PRJEB34838 | Wee et al., 2020 |
|  | *Lepidotrigona* | *ventralis* | [PRJNA387986](https://www.ncbi.nlm.nih.gov/bioproject/PRJNA387986) | Chen et al., 2017 |
|  | *Melipona* | *quadrifasciata* | [PRJNA279820](https://www.ncbi.nlm.nih.gov/bioproject/PRJNA279820) | Kapheim et al., 2015 |
|  | *Tetragonula* | *mellipes* | [PRJNA579203](https://www.ncbi.nlm.nih.gov/bioproject/PRJNA579203) | Hereward et al., 2020 |
|  | *Tetragonula* | *hockingsi* | PRJNA578994 | Hereward et al., 2020 |
|  | *Tetragonula* | *davenporti* | PRJNA578998 | Hereward et al., 2020 |
|  | *Tetragonula* | *clypearis* | PRJNA578946 | Hereward et al., 2020 |
|  | *Tetragonula* | *carbonaria* | PRJNA578948 | Hereward et al., 2020 |
| Colletidae | *Colletes* | *gigas* | PRJNA597580 | Zhou et al., 2020 |
| Halictidae | *Nomia* | *melanderi* | [PRJNA598198](https://www.ncbi.nlm.nih.gov/bioproject/PRJNA598198) | Kapheim et al., 2019 |
|  | *Dufourea* | *novaeangliae* | PRJNA311229 | Kapheim et al., 2015 |
|  | *Lasioglossum* | *albipes* | [PRJNA174755](https://www.ncbi.nlm.nih.gov/bioproject/PRJNA174755) | Kocher et al., 2013 |
|  | *Megalopta* | *genalis* | PRJNA625282 | Kapheim et al., 2020 |
| Megachilidae | *Osmia* | *bicornis* | PRJNA542365 | Beadle et al., 2019 |
|  | *Osmia* | *lignaria* | PRJNA553797 | Melicher et al. 2020 |
|  | *Megachile* | *rotundata* | PRJNA66515 | Kapheim et al., 2015 |

REFERENCES

Beadle, K., Singh, K. S., Troczka, B. J., Randall, E., Zaworra, M., Zimmer, C. T., Hayward, A., Reid, R., Kor, L., & Kohler, M. (2019). Genomic insights into neonicotinoid sensitivity in the solitary bee *Osmia bicornis*. PLoS Genetics, 15(2), e1007903.

Brand, P., Saleh, N., Pan, H., Li, C., Kapheim, K. M., & Ramírez, S. R. (2017). The nuclear and mitochondrial genomes of the facultatively eusocial orchid bee *Euglossa dilemma*. G3: Genes, Genomes, Genetics, 7(9), 2891-2898.

de Paula Freitas, F. C., Lourenço, A. P., Nunes, F. M., Paschoal, A. R., Abreu, F. C., Barbin, F. O., Bataglia, L., Cardoso-Júnior, C. A., Cervoni, M. S., & Silva, S. R. (2020). The nuclear and mitochondrial genomes of *Frieseomelitta varia*–a highly eusocial stingless bee (Meliponini) with a permanently sterile worker caste. BMC Genomics, 21, 1-26.

Heraghty, S. D., Sutton, J. M., Pimsler, M. L., Fierst, J. L., Strange, J. P., & Lozier, J. D. (2020). De Novo Genome Assemblies for Three North American Bumble Bee Species: *Bombus bifarius, Bombus vancouverensis,* and *Bombus vosnesenskii*. G3: Genes, Genomes, Genetics, 10(8), 2585-2592.

Hereward, J.P., Smith, T.J., Brookes, D.R. and Walter, G.H. (2020). Direct Submission to NCBI

Kapheim, K. M., Jones, B. M., Pan, H., Li, C., Harpur, B. A., Kent, C. F., Zayed, A., Ioannidis, P., Waterhouse, R. M., & Kingwell, C. (2020). Developmental plasticity shapes social traits and selection in a facultatively eusocial bee. Proceedings of the National Academy of Sciences, 117(24), 13615-13625.

Kapheim, K. M., Pan, H., Li, C., Blatti III, C., Harpur, B. A., Ioannidis, P., Jones, B. M., Kent, C. F., Ruzzante, L., & Sloofman, L. (2019). Draft genome assembly and population genetics of an agricultural pollinator, the solitary alkali bee (Halictidae: *Nomia melanderi*). G3: Genes, Genomes, Genetics, 9(3), 625-634.

Kapheim, K. M., Pan, H., Li, C., Salzberg, S. L., Puiu, D., Magoc, T., Robertson, H. M., Hudson, M. E., Venkat, A., & Fischman, B. J. (2015). Genomic signatures of evolutionary transitions from solitary to group living. Science, 348(6239), 1139-1143.

Kocher, S. D., Li, C., Yang, W., Tan, H., Soojin, V. Y., Yang, X., Hoekstra, H. E., Zhang, G., Pierce, N. E., & Douglas, W. Y. (2013). The draft genome of a socially polymorphic halictid bee, *Lasioglossum albipes*. Genome Biology, 14(12), 1-14.

Lin, D., Lan, L., Zheng, T., Shi, P., Xu, J. and Li, J. (2020). Direct Submission to NCBI.

Melicher, D., Rinehart, J.P., Yocum, G.D., Puppo, P. and Bowsher, J.H. (2020). Direct Submission to NCBI.

Nguyen, L., Nguyen, N., Okwuonu, G., Ongeri, F., Pham, C., Reid, J.G., Rio Deiros, D., Santibanez, J., Wang, M., Wu, Y.-Q., Scherer, S., Newsham, I., Worley, K.C., Muzny, D.M. and Gibbs, R. (2019). Direct Submission to NCBI

Oppenheim, S., Cao, X., Rueppel, O., Krongdang, S., Phokasem, P., DeSalle, R., Goodwin, S., Xing, J., Chantawannakul, P., & Rosenfeld, J. A. (2020). Whole genome sequencing and assembly of the Asian honey bee *Apis dorsata*. Genome biology evolution, 12(1), 3677-3683.

Park, D., Jung, J. W., Choi, B.-S., Jayakodi, M., Lee, J., Lim, J., Yu, Y., Choi, Y.-S., Lee, M.-L., & Park, Y. (2015). Uncovering the novel characteristics of Asian honey bee, *Apis cerana*, by whole genome sequencing. BMC Genomics, 16(1), 1-16.

Rehan, S. M., Glastad, K. M., Lawson, S. P., & Hunt, B. G. (2016). The genome and methylome of a subsocial small carpenter bee, *Ceratina calcarata*. Genome biology evolution, 8(5), 1401-1410.

Rehan, S. M., Glastad, K. M., Steffen, M. A., Fay, C. R., Hunt, B. G., & Toth, A. L. (2018). Conserved genes underlie phenotypic plasticity in an incipiently social bee. Genome biology evolution, 10(10), 2749-2758.

Sadd, B. M., Barribeau, S. M., Bloch, G., De Graaf, D. C., Dearden, P., Elsik, C. G., Gadau, J., Grimmelikhuijzen, C. J., Hasselmann, M., & Lozier, J. D. (2015). The genomes of two key bumblebee species with primitive eusocial organization. Genome Biology, 16(1), 1-32.

Shell WA, Steffen MA, Pare HK, Seetharam AS, Severin AJ, Toth AL, Rehan SM, 2021. Sociality sculpts similar patterns of molecular evolution in two independently evolved lineages of eusocial bees. Communications Biology 4:1-9.

Sun, C., Huang, J., Wang, Y., Zhao, X., Su, L., Thomas, G. W., Zhao, M., Zhang, X., Jungreis, I., & Kellis, M. (2021). Genus-wide characterization of bumblebee genomes provides insights into their evolution and variation in ecological and behavioral traits. Molecular biology evolution, 38(2), 486-501.

Qu, J., Richards, S., Aqrawi, P., Blankenburg, K., Chen, D., Goodspeed, R., Gross, S., Holder, M., Jackson, L., Javaid, M., Joshi, V., Kovar, C., Lee, S., Mandapat, C., Mata, R., Mathew, T., Ngo, R., Chen, Y.L.S., Chen, Y.T., Su, K.Y. and Yu, C.Y. (2017). Direct Submission to NCBI

Wallberg, A., Bunikis, I., Pettersson, O. V., Mosbech, M.-B., Childers, A. K., Evans, J. D., Mikheyev, A. S., Robertson, H. M., Robinson, G. E., & Webster, M. T. (2019). A hybrid de novo genome assembly of the honeybee, *Apis mellifera*, with chromosome-length scaffolds. BMC Genomics, 20(1), 275.

Wee, C.-Y., Tamizi, A.-A., Nazaruddin, N.-H., Ng, S.-M., Khoo, J.-S., & Jajuli, R. (2020). First Draft Genome Assembly of the Malaysian Stingless Bee, *Heterotrigona itama* (Apidae, Meliponinae). Data, 5(4), 112.

Zhou, Q.-S., Luo, A., Zhang, F., Niu, Z.-Q., Wu, Q.-T., Xiong, M., Orr, M. C., & Zhu, C.-D. (2020). The first draft genome of the plasterer bee *Colletes gigas* (Hymenoptera: Colletidae: Colletes). Genome biology evolution, 12(6), 860-866.
